# Pore-Forming Protein LIN-24 Enhances Starvation Resilience in *Caenorhabditis elegans* by Modulating Lipid Metabolism and Mitochondrial Dynamics

**DOI:** 10.3390/toxins17020072

**Published:** 2025-02-06

**Authors:** Xinqiang Lan, Mengqi Yang, Jiali Wang, Chunping Huang, Andong Wu, Leilei Cui, Yingqi Guo, Lin Zeng, Xiaolong Guo, Yun Zhang, Yang Xiang, Qiquan Wang

**Affiliations:** 1 Metabolic Control and Aging, Human Aging Research Institute and School of Life Science, Nanchang University, Jiangxi Key Laboratory of Human Aging, Nanchang 330031, China; lanxinqiang@ncu.edu.cn (X.L.); 405600220046@email.ncu.edu.cn (M.Y.); wangjiali@email.ncu.edu.cn (J.W.); chunpinghuang@email.ncu.edu.cn (C.H.); andongwu@ncu.edu.cn (A.W.); leileicui_xuan@hotmail.com (L.C.); 2 Institutional Center for Shared Technologies and Facilities of the Kunming Institute of Zoology, Chinese Academy of Sciences, Kunming 650204, China; guoyingqi@mail.kiz.ac.cn (Y.G.); zlkmzo@mail.kiz.ac.cn (L.Z.); 3 School of Physical Education, Yunnan Normal University, Kunming 650500, China; guoxiaolong@ynnu.edu.cn; 4 Key Laboratory of Animal Models and Human Disease Mechanisms of the Chinese Academy of Sciences/Key Laboratory of Bioactive Peptides of Yunnan Province, Kunming Institute of Zoology, The Chinese Academy of Sciences, Kunming 650204, China; zhangy@mail.kiz.ac.cn

**Keywords:** starvation, pore-forming protein, LIN-24, fatty acid degradation, skeletal muscle, donut-shaped mitochondria

## Abstract

The ability to survive starvation is a critical evolutionary adaptation, yet the molecular mechanisms underlying this capability remain incompletely understood. Pore-forming proteins (PFPs) are typically associated with immune defense, where they disturb the membranes of target cells. However, the role of PFPs in non-immune functions, particularly in metabolic and structural adaptations to starvation, is less explored. Here, we investigate the aerolysin-like PFP LIN-24 in *Caenorhabditis elegans* and uncover its novel function in enhancing starvation resistance. We found that LIN-24 expression is upregulated during starvation, leading to increased expression of the lipase-encoding gene *lipl-3*. This upregulation accelerates the mobilization and degradation of lipid stores, thereby sustaining energy levels. Additionally, LIN-24 overexpression significantly preserves muscle integrity, as evidenced by the maintenance of muscle structure compared to wild-type worms. Furthermore, we demonstrate that LIN-24 induces the formation of donut-shaped mitochondria, a structural change likely aimed at reducing ATP production to conserve energy during prolonged nutrient deprivation. This mitochondrial remodeling depends on genes involved in mitochondrial dynamics, including *mff-1*, *mff-2*, *drp-1*, and *clk-1*. Collectively, these findings expand our understanding of PFPs, demonstrating their multifaceted role in stress resistance beyond immune defense. LIN-24’s involvement in regulating metabolism, preserving muscle structure, and remodeling mitochondria highlights its crucial role in the adaptive response to starvation, offering novel insights into the evolution of stress resistance mechanisms and potential therapeutic targets for conditions related to muscle preservation and metabolic regulation.

## 1. Introductions

Survival in the natural world requires organisms to overcome numerous challenges, one of the most pervasive being starvation [1,2]. Throughout evolutionary history, both lower and higher animals have developed a diverse array of mechanisms and molecules to combat the detrimental effects of hunger [3,4,5]. These adaptations are crucial for survival during periods of food scarcity, allowing organisms to maintain vital functions and cellular integrity. For example, some animals enter states of dormancy or metabolic slowdown, reducing energy consumption to survive prolonged periods without food [6]. Others have evolved specific proteins and enzymes that enhance the efficiency of nutrient utilization or mobilize stored energy reserves [7]. Despite significant progress in understanding these mechanisms, the full spectrum of how animals resist starvation remains largely uncharted. Much of the current knowledge is derived from studies on metabolic pathways, hormonal regulation, and behavioral adaptations, but the role of specific molecular players in this complex process continues to be a fertile area of research.

Aerolysins are bacterial β-pore-forming toxins known for their ability to form pores in cellular membranes [8]. Proteins with an aerolysin fold, referred to as aerolysin family pore-forming proteins (af-PFPs), are found in various animal and plant species [8,9,10,11]. These proteins are well-recognized for their roles in microbial defense, where they disturb the integrity of target cell membranes by creating pores [12]. Our recent research has focused on the af-PFP βγ-CAT from the toad *Bombina maxima* [13]. This af-PFP is negatively regulated by its paralog BmALP3 in an oxygen-dependent manner, allowing it to exist in active or inactive forms [14]. Upon activation, βγ-CAT targets acidic glycosphingolipids in lipid rafts, leading to the endocytosis of its α-subunit BmALP1 [11]. This process stimulates pinocytosis and forms channels along the endolysosomal pathway, altering intracellular vesicle contents and modulating exocytosis, including the release of functional EVs, thereby contributing to immune defense [11,15,16]. Interestingly, we recently demonstrated that βγ-CAT can protect cells from starvation-induced cell death in vitro [17,18]. This discovery opens up intriguing possibilities regarding the broader biological functions of af-PFPs. However, it remains unclear whether these proteins can confer starvation resistance at the level of the whole organism. This question is particularly pertinent given the complexity of starvation responses, which involve a coordinated interplay between cellular, tissue, and systemic processes [18,19,20].

To address this gap, we turned to *Caenorhabditis elegans*, a model organism that offers numerous advantages for biological research [21,22], including the study of starvation [23]. The simplicity of its anatomy, its well-characterized genetics, and the conservation of many biological pathways make *C. elegans* an ideal system for investigating fundamental biological questions [22,24]. In previous studies, we demonstrated that LIN-24, an af-PFP, plays a critical role in enhancing the survival of *C. elegans* under microbial infection conditions. LIN-24 achieves this by upregulating genes involved in immune and defense responses, maintaining lysosomal function, and activating the transcription factor DAF-16, which in turn drives the expression of antibacterial genes such as *dod-22*. This activation of the immune response helps the worms effectively combat bacterial infections [25]. In the current study, through knockdown and overexpression experiments, we found that LIN-24 significantly enhances the survival of *C. elegans* under starvation conditions. Mechanistically, we discovered that LIN-24 upregulates genes involved in fatty acid metabolism, preserves skeletal muscle integrity, and promotes the formation of donut-shaped mitochondria, which are associated with increased stress resistance during starvation. The findings from our study highlight a novel function for af-PFPs in starvation resistance at the organismal level. The ability of LIN-24 to enhance starvation survival in *C. elegans* provides a compelling example of how these proteins may contribute to adaptive stress responses. This work not only broadens our understanding of PFPs but also sheds light on the intricate strategies employed by organisms to withstand starvation.

## 2. Results

### 2.1. LIN-24 in C. elegans Is Upregulated in Response to Starvation and May Play a Role in Fatty Acid Metabolism

**Figure 1:**
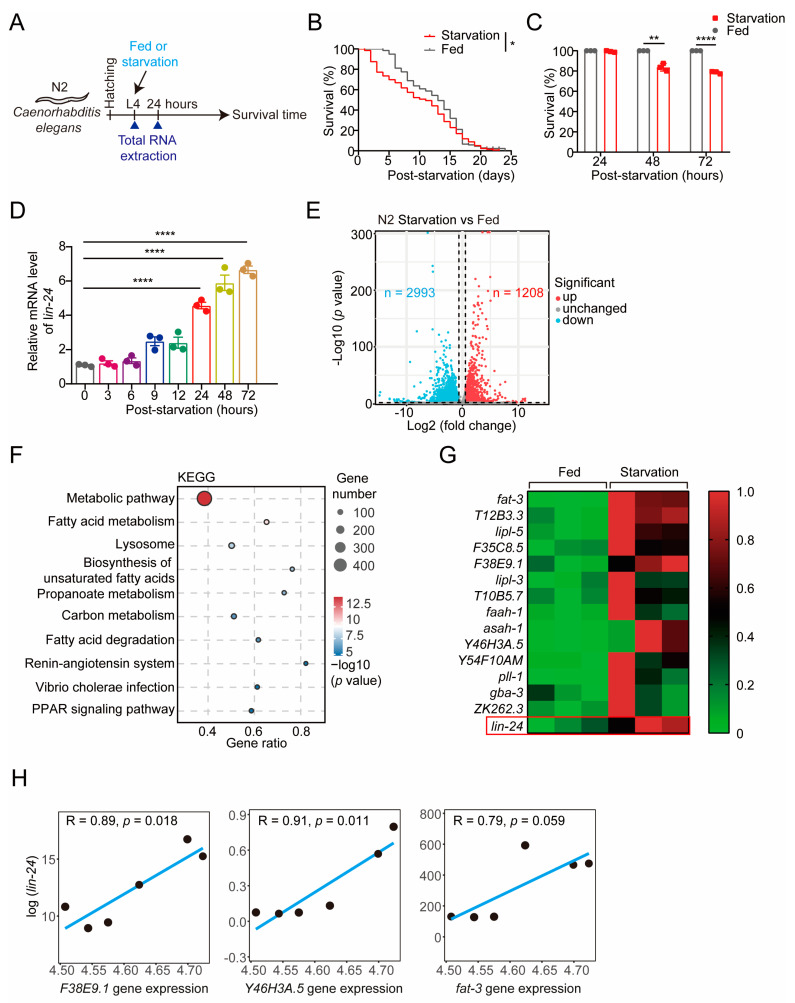
Upregulation of LIN-24 in *C. elegans* in response to starvation. (**A**) A schematic diagram illustrating the experimental design of the *C. elegans* starvation model. (**B**) A lifespan analysis of worms under normal feeding and starvation conditions (*n* = 136 for the normal feeding group and *n* = 99 for the starvation group). (**C**) The survival rates of worms under normal feeding conditions and after 24, 48, and 72 h of starvation (*n* = 3). (**D**) A quantitative PCR (qPCR) analysis of *lin-24* mRNA levels in worms subjected to various durations of starvation (*n* = 3). (**E**) A volcano plot showing differentially expressed genes (DEGs) identified by RNA-seq in worms under normal feeding and after 24 h of starvation. Genes with *p*-values < 0.05 and fold changes >1.5 were considered significantly changed (red: upregulated; blue: downregulated). (**F**) A KEGG pathway enrichment analysis of differentially expressed genes in worms under normal feeding and after 24 h of starvation. The top 10 enriched pathways are shown. (**G**) A heatmap of genes related to the fatty acid degradation pathway identified in panels (**E**,**F**) (*n* = 3). (**H**) A Pearson correlation analysis of the expression levels of *F38E9.1*, *Y46H3A.5*, and *fat-3* with *lin-24* in worms. The correlation coefficients (R) and significance levels (*p*) are shown. Data in bar graphs are expressed as mean ± SD, and significance was determined using an unpaired two-tailed *t*-test (**C**) or one-way ANOVA followed by Tukey’s post hoc test (**D**). ** *p* < 0.01 and **** *p* < 0.0001 vs. fed. Survival curves were plotted using Kaplan–Meier survival analysis, and significance was estimated by log-rank test. * *p* < 0.05 vs. fed.

To investigate the role of the PFP LIN-24 in the starvation response of *C. elegans*, we established a starvation model by depriving worms of food following the L4 larval stage, while maintaining a control group under normal feeding conditions (Figure 1A). Survival analysis revealed a marked increase in mortality in starved worms compared to the control group, particularly evident after 48 h of food deprivation (Figure 1B,C). Given our previous findings that the PFP βγ-CAT from *Bombina maxima* is upregulated during starvation [17], we hypothesized that LIN-24 may similarly be involved in the starvation response of *C. elegans*. qPCR analysis supported this hypothesis, showing a significant upregulation of LIN-24 mRNA levels after 24 h of starvation (Figure 1D), indicating a potential role in the organism’s response to nutrient deprivation.

To further elucidate the function of LIN-24 during starvation, we performed transcriptome sequencing on *C. elegans* subjected to 24 h of starvation and on normally fed controls. The transcriptomic analysis identified 1208 genes that were significantly upregulated and 2993 genes that were significantly downregulated in response to starvation (Figure 1E and Table S1). Consistent with findings in other organisms, KEGG (Kyoto Encyclopedia of Genes and Genomes) pathway analysis revealed the significant enrichment of differentially expressed genes (DEGs) in metabolic pathways (Figure 1F), particularly those associated with fatty acid degradation (Figure 1F,G). Moreover, transcriptomic data corroborated the qPCR results, confirming the upregulation of *lin-24* mRNA following 24 h of starvation (Figure 1G). Gene correlation analysis indicated that *lin-24* expression during starvation was positively correlated with the expression of lipid metabolism-related genes *F38E9.1* (lipid metabolic process, ALLIANCE of Genome RESOURCES) and *Y46H3A.5* (lipid metabolic process, ALLIANCE of Genome RESOURCES), and marginally positively correlated with *fat-3* (biosynthesis of unsaturated fatty acids, ALLIANCE of Genome RESOURCES) [26,27] (Figure 1H). These findings suggest that LIN-24 may play a significant role in the adaptive response to starvation, potentially through the regulation of fatty acid metabolism in *C. elegans*.

### 2.2. LIN-24 Overexpression Enhances Starvation Resistance in C. elegans

**Figure 2:**
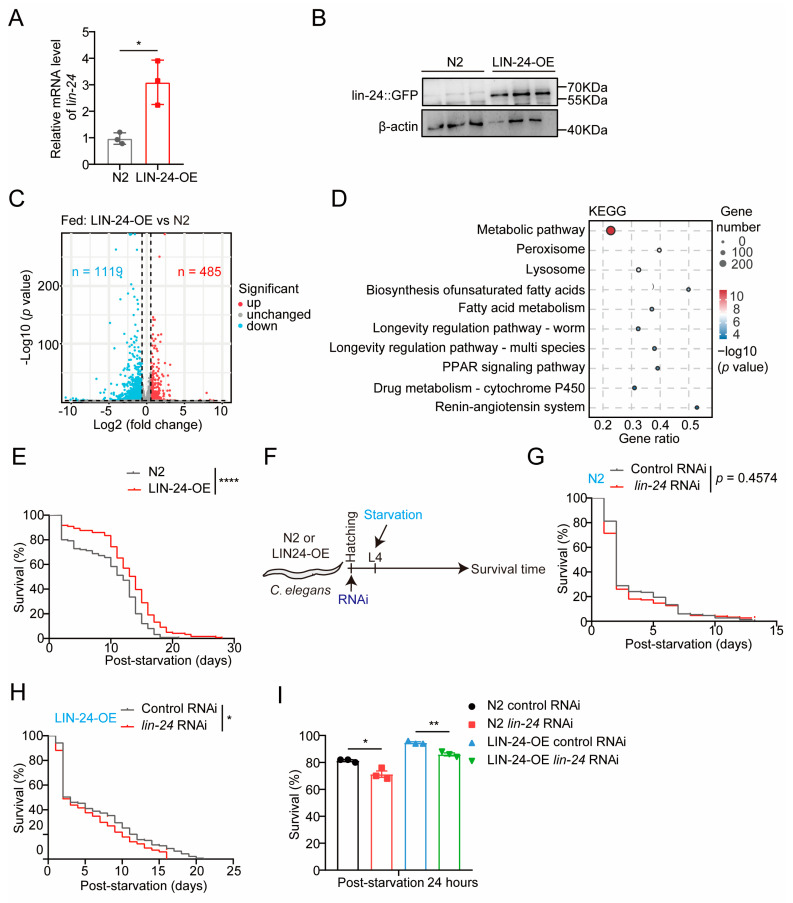
LIN-24 overexpression enhances starvation resistance in *C. elegans*. (**A**) *lin-24* mRNA levels in N2 and LIN-24-OE worms, as detected by qPCR. (**B**) LIN-24 protein levels in N2 and LIN-24-OE worms, as detected by Western blotting using anti-GFP antibody. (**C**) Volcano plot showing DEGs identified by RNA-seq in N2 and LIN-24-OE worms under normal feeding conditions. Genes with *p*-values < 0.05 and fold changes >1.5 were considered significantly changed (red: upregulated; blue: downregulated). (**D**) KEGG pathway enrichment analysis of differentially expressed genes in N2 and LIN-24-OE worms under normal feeding conditions. Top 10 enriched pathways are shown. (**E**) Lifespan analysis of N2 and LIN-24-OE worms under starvation conditions (*n =* 125 for N2 group and *n =* 121 for LIN-24-OE group). (**F**) Schematic diagram illustrating experimental design of LIN-24 RNAi worms in starvation model. (**G**) Lifespan analysis of wild-type N2 worms with LIN-24 RNAi under starvation conditions (*n =* 149 for control RNAi group and *n =* 150 for LIN-24 RNAi group). (**H**) Lifespan analysis of LIN-24-OE worms with LIN-24 RNAi under starvation conditions (*n =* 139 for control RNAi group and *n =* 178 for LIN-24 RNAi group). (**I**) Survival rates of worms from panels (**G**,**H**) following 24 h of starvation. Data in bar graphs are expressed as mean ± SD and significance was determined using unpaired two-tailed *t*-test. * *p* < 0.05 and ** *p* < 0.01 vs. N2. Survival curves were plotted using Kaplan–Meier survival analysis, and significance was estimated by log-rank test. **** *p* < 0.0001 vs. Fed. * *p* < 0.05 vs. control RNAi.

To explore the functions of LIN-24 in the starvation response of *C. elegans*, we conducted starvation experiments using LIN-24 overexpression (LIN-24-OE) worms that were previously generated in our laboratory [25]. The successful overexpression of LIN-24 was confirmed by both qPCR and Western blot analyses, which showed significant increases in *lin-24* mRNA and protein levels compared to wild-type (N2) controls (Figure 2A,B). These validations ensured the robustness of our LIN-24-OE model for subsequent experiments. We next performed transcriptome sequencing on LIN-24-OE worms and N2 controls under normal feeding conditions. The analysis revealed that LIN-24 overexpression significantly upregulated 485 genes and downregulated 119 genes (Figure 2C and Table S2). KEGG pathway analysis revealed significant enrichment of the DEGs involved in starvation-related pathways, including metabolic pathways and fatty acid metabolism (Figure 2D). To further explore the transcriptional changes induced by LIN-24 overexpression, we performed a comparative analysis of DEGs between LIN-24-OE worms and N2 controls under normal feeding and starvation conditions. The results showed substantial overlap between the genes altered by LIN-24 overexpression and those differentially expressed in N2 worms during starvation, including genes involved in metabolic pathways and fatty acid metabolism (Figure S1 and Table S3). These findings suggest that LIN-24 likely plays a pivotal role in enhancing starvation resistance in *C. elegans*. Further functional assays supported this hypothesis, as we observed a significant increase in the survival rate of LIN-24-OE worms under starvation conditions (Figure 2E). To further confirm these findings, we conducted RNA interference (RNAi) experiments targeting LIN-24 (Figure 2F). While LIN-24 RNAi in wild-type N2 worms did not significantly affect overall survival, it notably reduced the survival rate after 24 h of starvation (Figure 2G,I). Additionally, RNAi-mediated knockdown of LIN-24 in the LIN-24-OE worms resulted in a significant reduction in both overall survival and survival after 24 h of starvation (Figure 2H,I). These results strongly suggest that LIN-24 plays a protective role in helping *C. elegans* resist starvation.

### 2.3. LIN-24 Promotes Fatty Acid Metabolism During Starvation

**Figure 3:**
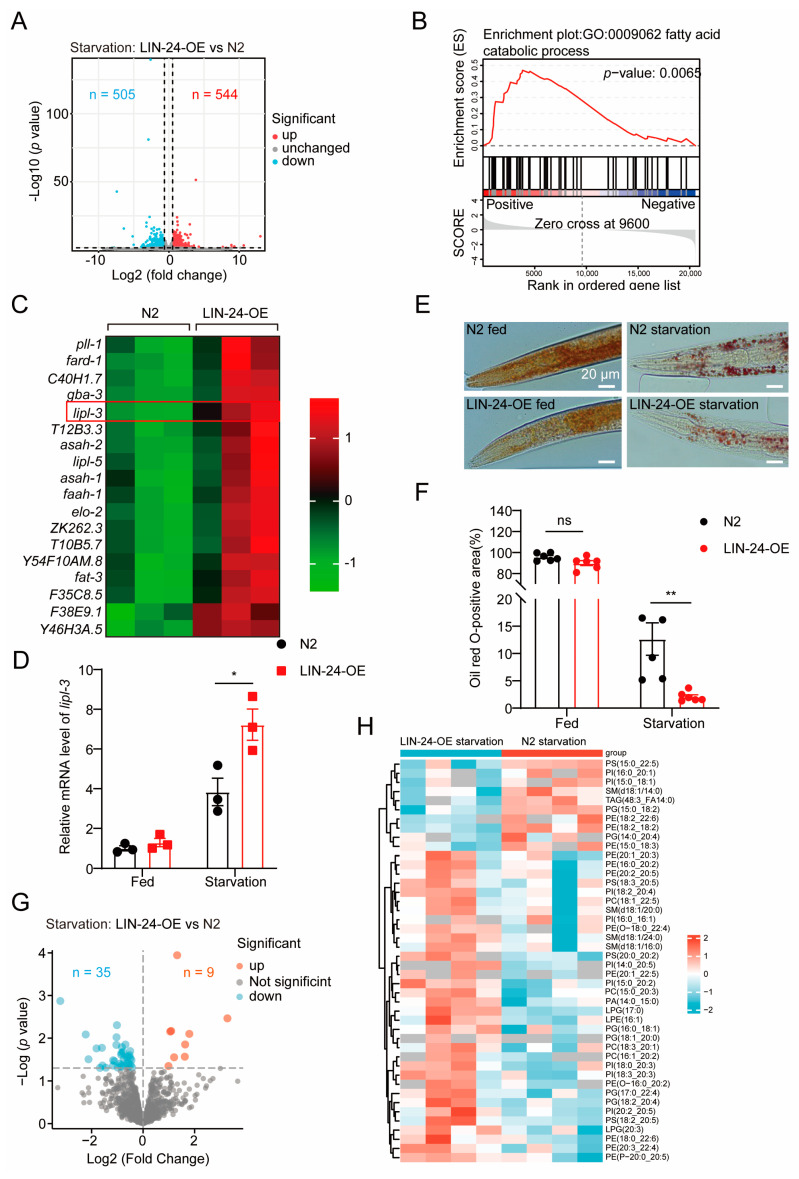
LIN-24 overexpression promotes fatty acid metabolism during starvation. (**A**) Volcano plot showing DEGs identified by RNA-seq in N2 and LIN-24-OE worms after 24 h of starvation. Genes with *p*-values < 0.05 and fold changes >1.5 were considered significantly changed (red: upregulated; blue: downregulated). (**B**) GSEA pathway enrichment analysis indicates that LIN-24-OE worms are involved in processes related to fatty acid catabolic pathways. (**C**) Heatmap of genes related to fatty acid metabolism and degradation identified in panels (**A**,**B**) (*n* = 3). (**D**) qPCR analysis of *lipl-3* mRNA levels in N2 and LIN-24-OE worms subjected to starvation (*n* = 3). (**E**) Oil Red O staining and quantification (**F**) in N2 and LIN-24-OE worms under both fed and starved conditions (*n* = 5–6). Scale bar, 20 μm. (**G**) Volcano plot showing differential expression of lipids between LIN-24-OE and N2 worms during starvation. (**H**) Heatmap of lipid species identified in LIN-24-OE and N2 worms under starvation conditions. Data are expressed as mean ± SD. Significance was determined using unpaired two-tailed *t*-test. * *p* < 0.05; ** *p* < 0.01; ns = not significant.

To investigate how LIN-24 contributes to starvation resistance in *C. elegans*, we analyzed the transcriptomes of N2 wild-type worms and LIN-24-OE worms after 24 h of starvation. Compared to control N2 worms, LIN-24-OE worms exhibited significant upregulation of 544 genes and downregulation of 505 genes (Figure 3A and Table S4). During starvation, animals typically enhance the expression of genes involved in fatty acid metabolism, especially for fatty acid degradation [20,28,29,30,31], as the breakdown of fatty acids provides a critical energy source. Previous studies have shown that the upregulation of fatty acid degradation-related genes is essential for surviving starvation [32,33]. Consistent with the observed correlation between LIN-24 and genes related to fatty acid degradation during starvation (Figure 1H), Gene Set Enrichment Analysis (GSEA) indicated that LIN-24 overexpression positively regulates the fatty acid catabolic process (Figure 3B,C).

We also compared gene expression changes in LIN-24-OE worms under both starvation and normal feeding conditions, finding that LIN-24 overexpression significantly influences fatty acid metabolism in both scenarios, further emphasizing its role in regulating lipid metabolism under nutritional stress (Figure S2 and Table S5). One gene of particular interest was *lipl-3*, which was significantly upregulated in LIN-24-OE worms under starvation conditions (Figure 3C,D). *lipl-3* encodes a lipase enzyme that is crucial for lipid metabolism [34], specifically for breaking down stored fat into free fatty acids [34,35]. These free fatty acids can then be used as an energy source [34]. The upregulation of *lipl-3* in response to LIN-24 overexpression suggests that LIN-24 enhances the breakdown of fatty acids, thereby supplying the energy needed for *C. elegans* to survive during periods of nutrient scarcity. To confirm this, we performed Oil Red O staining to assess fat storage in N2 and LIN-24-OE worms under both fed and starved conditions. The results were consistent with the transcriptomic data, showing that LIN-24-OE worms had significantly reduced fat stores after 24 h of starvation compared to N2 worms (Figure 3E,F).

To further validate the role of LIN-24 in lipid metabolism and starvation resistance, we supplemented our study with additional experiments. LC-MS/MS analysis revealed that under starvation conditions, LIN-24-OE worms exhibited a significant downregulation of 35 lipid molecules and an upregulation of 9 lipid molecules compared to N2 controls, suggesting that LIN-24 overexpression accelerates lipid degradation to meet energy demands (Figure 3G,H and Table S6). This reduction in fat stores indicates that LIN-24 overexpression accelerates fat mobilization, likely by upregulating fatty acid degradation pathways, to provide energy during starvation.

### 2.4. LIN-24 Modulates Amino Acid Metabolism and Preserves Skeletal Muscle Integrity During Starvation

**Figure 4:**
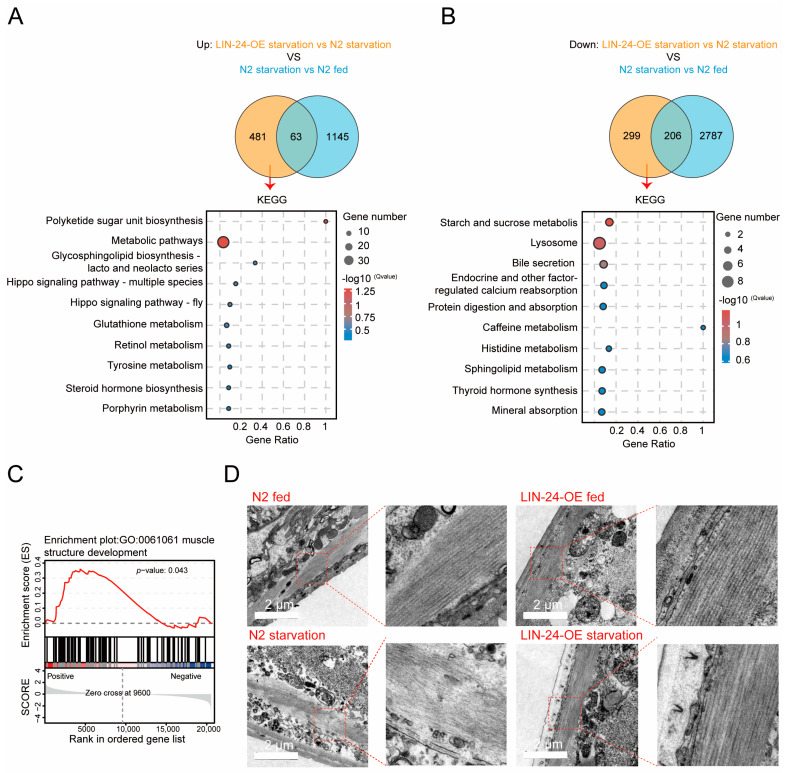
LIN-24 overexpression modulates amino acids metabolism and preserves muscle integrity during starvation. (**A**,**B**) Venn diagram analysis of gene expression in LIN-24-OE worms and N2 wild-type worms under starvation conditions. Venn diagram shows that 481 genes are uniquely upregulated in LIN-24-OE worms. KEGG pathway analysis of these upregulated genes is shown below, highlighting pathways such as polyketide sugar unit biosynthesis, metabolic pathways, and amino acid metabolism (**A**). Venn diagram shows that 299 genes are uniquely downregulated in LIN-24-OE worms. KEGG pathway analysis of these downregulated genes reveals significant enrichment in pathways related to starch and sucrose metabolism, lysosome function, bile secretion, and protein digestion and absorption (**B**). (**C**) GSEA pathway enrichment analysis suggesting that LIN-24-OE worms are implicated in processes related to muscle structure development during starvation. (**D**) Transmission electron microscopy (TEM) images showing morphology of body wall muscles in N2 and LIN-24-OE worms under both fed and starved conditions. Scale bar, 2 μm.

Skeletal muscle plays a critical role in overall body function [36,37,38], constituting approximately 40% to 50% of body mass and serving as the most protein-rich organ in animals [36,39,40]. Maintaining the structure and function of skeletal muscle is particularly important during starvation, as muscle tissue is a major source of amino acids that can be used for energy production when other energy reserves are depleted [41,42]. In this study, we compared the gene expression profiles of LIN-24-OE worms and N2 worms under starvation conditions using Venn diagram analysis to identify overlapping and unique gene expression changes. Our results revealed a significant overlap in gene expression between the two groups, with 63 genes upregulated and 206 genes downregulated in both LIN-24-OE and N2 worms. Additionally, 481 genes were uniquely upregulated, and 299 genes were uniquely downregulated in LIN-24-OE worms under starvation conditions (Figure 4A,B and Table S7). These unique genes were primarily involved in metabolic pathways beyond fatty acid metabolism, particularly in modulating amino acid metabolism and downregulating protein digestion and absorption (Figure 4A,B), suggesting that LIN-24 overexpression impacts gene expression in both overlapping and divergent ways when compared to the response seen in N2 worms. This finding implies that LIN-24 utilizes multiple pathways to enhance starvation resistance.

Further analysis using GSEA indicated that LIN-24 overexpression positively regulates muscle structure development during starvation (Figure 4C). Based on these findings, we hypothesized that LIN-24 may help preserve muscle integrity during nutrient deprivation. To test this, we examined the skeletal muscle structure of N2 and LIN-24-OE worms after 24 h of starvation using electron microscopy. The electron microscopy analysis revealed notable differences in muscle morphology between the two groups. In N2 worms, starvation led to significant muscle degradation, characterized by disrupted sarcomere organization, reduced muscle fiber thickness, and an overall loss of muscle integrity. In contrast, LIN-24-OE worms displayed well-preserved muscle structures, with sarcomeres that remained organized and muscle fibers that retained their thickness, even after 24 h of starvation (Figure 4D). This suggests that LIN-24 may contribute to starvation resistance by preserving skeletal muscle integrity and function.

### 2.5. LIN-24 Promotes Donut-Shaped Mitochondria Formation to Enhance Starvation Resistance in C. elegans

**Figure 5:**
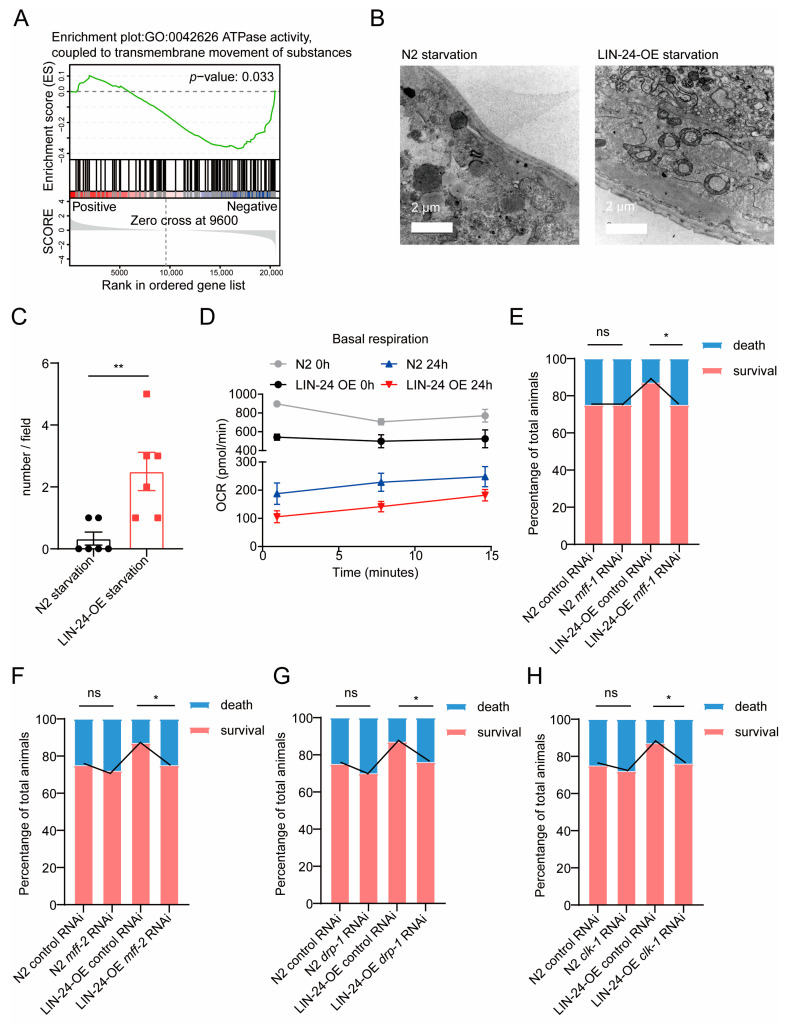
LIN-24 facilitates formation of donut-shaped mitochondria, enhancing *C. elegans’* resistance to starvation. (**A**) GSEA pathway enrichment analysis indicating that LIN-24 negatively regulates ATPase activity during starvation. (**B**) TEM images and quantification (**C**) showing morphology of donut-shaped mitochondria in N2 and LIN-24-OE worms during starvation (*n* = 6). Scale bar, 2 μm. (**D**) Basal respiration measurements of LIN-24 OE and N2 worms under normal feeding and starvation conditions using Seahorse assays. (**E**–**H**) Survival rates of N2 and LIN-24-OE worms following 48 h period of starvation after knocking down *mff-1*, *mff-2*, *drp-1*, and *clk-1*. Data in bar graphs are expressed as mean ± SD, and significance was determined using unpaired two-tailed *t*-test. ** *p* < 0.01 vs. N2 starvation. Survival curves were plotted using Kaplan–Meier survival analysis, and significance was estimated by log-rank test. * *p* < 0.05; ns = not significant.

Mitochondria are central hubs of energy metabolism [43], and the maintenance of mitochondrial structure and function is crucial for cellular survival, especially under conditions of nutrient scarcity [30,43]. Efficient energy conservation and mitochondrial remodeling have been demonstrated as effective strategies for organisms to resist starvation [44,45,46]. The above transcriptomic analysis and functional assays indicate that LIN-24 is involved in metabolic processes, particularly in fatty acid metabolism, during starvation in *C. elegans*. This suggests that LIN-24’s role in starvation resistance may be closely linked to mitochondrial function. Interestingly, the GSEA results also indicate that LIN-24 negatively regulates ATPase activity during starvation (Figure 5A), further supporting the hypothesis that LIN-24 might be involved in maintaining mitochondrial energy production under these conditions. To explore this possibility, we performed electron microscopy analysis on the mitochondria of N2 and LIN-24-OE worms after 24 h of starvation. Intriguingly, we observed a significant formation of donut-shaped mitochondria in the LIN-24-OE worms during starvation (Figure 5B,C). Donut-shaped mitochondria are a distinct mitochondrial morphology characterized by a circular ring-like structure [25,46,47,48]. This unusual shape is typically associated with a cellular response to stress, particularly in conditions where energy needs to be conserved [25,46,49]. The formation of donut-shaped mitochondria is thought to reduce mitochondrial surface area, thereby decreasing ATP production and minimizing energy expenditure, which can help cells survive during periods of limited nutrient availability [48,50]. To further explore this idea, we conducted Seahorse assays to evaluate mitochondrial function. Our results demonstrated that LIN-24 overexpression led to reduced basal respiration both under normal feeding conditions and after 24 h of starvation (Figure 5D).

The increased presence of donut-shaped mitochondria in LIN-24-OE worms suggests that LIN-24 may facilitate the formation of these structures as a mechanism to enhance starvation resistance. To test this hypothesis, we conducted RNAi experiments targeting genes known to be involved in the formation of donut-shaped mitochondria, including *mff-1*, *mff-2*, *drp-1*, and *clk-1* [51,52]. Our results showed that knocking down these genes significantly reduced the ability of LIN-24-OE worms to survive under starvation conditions (Figure 5E–H). These findings strongly suggest that LIN-24 contributes to starvation resistance in *C. elegans* by promoting the formation of donut-shaped mitochondria. This mitochondrial remodeling likely helps to conserve energy during starvation, thereby enhancing the survival of the organism.

## 3. Discussion

**Figure 6:**
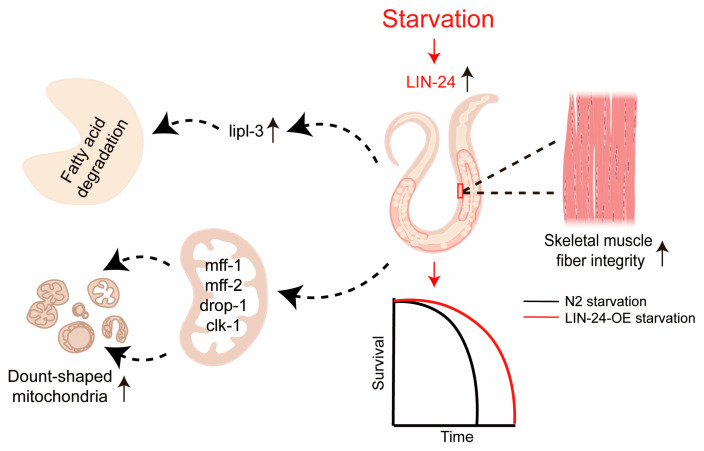
A model of LIN-24’s role in starvation resistance in *C. elegans*. During starvation, LIN-24 expression is upregulated, enhancing fat mobilization through the activation of the lipase gene *lipl-3*. This process supports energy homeostasis by promoting the degradation of fat stores. LIN-24 also plays a crucial role in maintaining muscle structure and integrity under nutrient deprivation. Additionally, LIN-24 induces the formation of donut-shaped mitochondria, which are linked to increased stress resistance and energy conservation by reducing ATP production. This mitochondrial remodeling is regulated by key genes involved in mitochondrial dynamics, including *mff-1*, *mff-2*, *drp-1*, and *clk-1*.

In this study, we have demonstrated that the af-PFP LIN-24 in *C. elegans* plays a critical role in enhancing starvation resistance. Our findings indicate that LIN-24 facilitates survival under nutrient scarcity by regulating key metabolic processes, including fatty acid metabolism, preserving skeletal muscle structure, and promoting mitochondrial remodeling (Figure 6). These results highlight a novel function for PFPs beyond their established role in immune defense, suggesting that LIN-24 is integral to the adaptive response to starvation. By exploring the molecular mechanisms underlying LIN-24’s function, we provide new insights into how *C. elegans* and potentially other organisms utilize PFPs to cope with periods of nutrient deprivation.

Our results show that LIN-24 is upregulated in response to starvation and promotes the expression of the lipase gene *lipl-3*, which is critical for the breakdown of fat stores [34,35]. The upregulation of *lipl-3* in LIN-24-OE worms correlates with a significant reduction in fat stores, as observed through Oil Red O staining. This suggests that LIN-24 enhances the mobilization and degradation of fatty acids, thereby supplying energy during periods of nutrient scarcity. These findings are consistent with previous studies, demonstrating the importance of fatty acid metabolism in the survival of starved organisms [20,29,30,32]. The accelerated breakdown of fat reserves in LIN-24-OE worms underscores the importance of LIN-24 in sustaining energy levels during starvation. This function of LIN-24 may be particularly advantageous in environments where food availability is unpredictable, providing a competitive edge by enabling prolonged survival without nutrient intake.

Our study also reveals that LIN-24 plays a crucial role in preserving skeletal muscle integrity during starvation. In LIN-24-OE worms, we observed well-maintained muscle structure and organization, even after 24 h of starvation, in contrast to the significant muscle degradation seen in wild-type worms. The preservation of muscle structure in LIN-24-OE worms is particularly notable given that muscle tissue is often one of the first to be catabolized during periods of nutrient deprivation to provide amino acids for energy production [41,42]. This suggests that LIN-24 may confer a protective effect on muscle tissue, potentially by modulating the balance between muscle protein synthesis and degradation during starvation. The ability to maintain muscle integrity during starvation is likely crucial for the overall fitness and survival of the organism [53,54], as it preserves muscle function for essential activities such as locomotion and feeding when nutrients become available again [55]. The mechanisms by which LIN-24 preserves muscle structure during starvation remain to be fully elucidated. It is possible that LIN-24 modulates signaling pathways involved in muscle maintenance, such as those regulating protein turnover or autophagy. Further studies are needed to investigate whether LIN-24 directly interacts with these pathways or if its protective effect on muscle tissue is mediated through other downstream effectors. Understanding these mechanisms could have broader implications for developing therapeutic strategies to prevent muscle wasting in various clinical contexts, including starvation, cachexia, and aging.

One of the most striking findings of this study is the role of LIN-24 in promoting the formation of donut-shaped mitochondria during starvation. Donut-shaped mitochondria are a distinct morphological adaptation associated with stress responses, particularly in conditions where energy conservation is critical [25,50]. In LIN-24-OE worms, we observed a significant increase in the formation of these structures under starvation conditions. The formation of donut-shaped mitochondria is thought to reduce mitochondrial surface area and, consequently, ATP production [50]. This reduction in energy output likely helps to conserve energy during nutrient deprivation, contributing to the organism’s ability to survive prolonged periods without food. Our RNAi experiments targeting genes involved in mitochondrial dynamics, such as *mff-1*, *mff-2*, *drp-1*, and *clk-1*, further support the role of LIN-24 in mitochondrial remodeling. The knockdown of these genes in LIN-24-OE worms significantly impaired their survival under starvation, indicating that the formation of donut-shaped mitochondria is a critical component of LIN-24’s function in enhancing starvation resistance. These findings suggest that LIN-24 may act as a regulator of mitochondrial dynamics, promoting structural changes that optimize energy use during starvation. This adds a new dimension to our understanding of how PFPs can influence cellular energy metabolism and stress responses.

The discovery of LIN-24’s role in starvation resistance has important implications for understanding the evolution of stress resistance mechanisms in organisms. PFPs, including those with aerolysin-like domains, have traditionally been studied in the context of immune defense, where they function by disturbing the membranes of target cells [8,56]. However, our findings suggest that PFPs have been co-opted for additional functions in stress resistance, particularly in the context of starvation. The ability of LIN-24 to regulate metabolism, preserve muscle structure, and remodel mitochondria highlights the versatility of PFPs as adaptive molecules that can be repurposed to meet the specific challenges posed by nutrient scarcity. This multifunctionality of PFPs may reflect an evolutionary advantage, allowing organisms to maximize the utility of these proteins across different physiological contexts. The co-option of immune-related proteins for roles in metabolism and stress resistance could represent a broader evolutionary strategy that has been conserved across diverse species. Further comparative studies across different organisms could provide insights into how PFPs have evolved to serve various functions, shedding light on the molecular underpinnings of stress resistance in both invertebrates and vertebrates.

The identification of LIN-24 as a key player in starvation resistance raises the potential for targeting this family of homologous PFPs or their downstream effectors for therapeutic purposes. In particular, LIN-24’s ability to maintain muscle integrity and promote mitochondrial remodeling during starvation could be significant for conditions involving muscle wasting, such as cachexia or age-related sarcopenia [37,57]. Mimicking these effects could potentially provide a means to protect muscle tissue and improve outcomes in these conditions. Additionally, LIN-24’s role in enhancing fatty acid metabolism suggests that this protein family might be explored as a target for metabolic disorders where energy balance is disrupted. By promoting the efficient mobilization and utilization of fat stores, LIN-24 or its analogs could help restore metabolic homeostasis. However, further research is needed to explore LIN-24’s potential as a therapeutic target, including studies on the function of this class of PFPs in higher organisms and their effects on different tissue types under various physiological conditions.

In this study, we observed that LIN-24 overexpression enhances fatty acid degradation, providing an important energy source during starvation. Fatty acids undergo β-oxidation to produce acetyl-CoA, which enters the TCA cycle and generates ATP to support cellular functions necessary for survival. Interestingly, LIN-24 also induces the formation of donut-shaped mitochondria, which reduces mitochondrial surface area. While this might suggest decreased ATP production, this morphological change likely represents an energy-saving mechanism. Reducing the mitochondrial surface area helps minimize energy expenditure during starvation, allowing mitochondria to remain efficient in energy production while conserving resources. These findings highlight a dual strategy in which LIN-24 enhances fatty acid breakdown for energy production and promotes mitochondrial changes that conserve energy, thus ensuring survival under nutrient-poor conditions. How LIN-24 coordinates these two processes will be an intriguing direction for future research.

In conclusion, our study reveals a novel role for the PFP LIN-24 in enhancing starvation resistance in *C. elegans*. By upregulating genes involved in fatty acid metabolism, preserving skeletal muscle integrity, and promoting the formation of donut-shaped mitochondria, LIN-24 plays a crucial role in the organism’s adaptive response to nutrient deprivation (Figure 6). These findings not only expand our understanding of LIN-24’s function beyond immune defense but also highlight its potential as a target for therapeutic strategies aimed at improving muscle preservation and metabolic resilience during starvation or related stress conditions. This work adds to the growing body of evidence that PFPs have diverse biological roles, offering new insights into the mechanisms that organisms use to survive in challenging environments.

## 4. Materials and Methods

### 4.1. C. elegans Strains and Culture

The *C. elegans* strains used in this study, including N2 Bristol (wild type) and the *Escherichia coli* OP50 strain (food source), were obtained from the Caenorhabditis Genetics Center (CGC). The strains overexpressing LIN-24 fused to green fluorescent protein (GFP) were generated at SunyBiotech [28]. All strains were derived from the Bristol N2 strain and were cultured at 20 °C on nematode growth media (NGM) agar plates seeded with *E. coli* OP50.

### 4.2. Starvation Resistance Assays

Synchronized L1 larvae were transferred to standard OP50 NGM plates. After 60 h, when the nematodes reached the L4 or early adult stage, they were washed off the plates with M9 buffer and collected in 15 mL centrifuge tubes. The tubes were centrifuged at 3000 rpm for 1 min at room temperature, and the supernatant was discarded. This washing step was repeated three times. The nematodes were then transferred to 12-well plates, with 50 nematodes per well in 2 mL of M9 buffer containing 100 µg/mL AMP and 50 µg/mL 5-FuDR (FuDR; Sigma, St Louis, MO, USA). The plates were incubated at 20 °C. Day 0 of the starvation survival assay was defined as the day the nematodes were transferred to the new plates. Nematodes were counted daily using a double-blind method and transferred to fresh M9 buffer containing AMP and 5-FuDR every day. Control animals were maintained on standard OP50 NGM agar plates supplemented with AMP and 5-FuDR and were not subjected to starvation conditions. Both the experimental and control groups were kept at 20 °C throughout the experiment.

### 4.3. RNA Interference Starvation Resistance Assay

Synchronized L1 larvae were transferred to NGM plates containing either L4440 control bacteria or RNA interference bacteria. Once they reached the L4 or early adult stage, the nematodes were washed off using M9 buffer and collected in 15 mL centrifuge tubes. The tubes were centrifuged at 3000 rpm for 1 min at room temperature and the supernatant was discarded. The washing step was repeated three times. The nematodes were then transferred to 12-well plates containing M9 buffer with 100 µg/mL AMP and 50 µg/mL 5-FuDR. Survival was monitored daily, and data were recorded for subsequent analysis.

### 4.4. RNA Isolation and qPCR

Samples were immersed in TRIzol reagent and homogenized using a tissue lyser (Servicebio, Wuhan, China). RNA extraction followed the manufacturer’s instructions (Invitrogen, Waltham, MA, USA; Thermo Fisher Scientific, San Francisco, CA, USA). RNA concentration and purity were determined by measuring optical density at 260 nm using a NanoDrop^®^ ND-2000 spectrophotometer(Thermo Fisher Scientific, San Francisco, CA, USA). RNA (1 μg) was reverse-transcribed using a PrimeScript™ RT reagent kit (TaKaRa) with a gDNA Eraser (TaKaRa) according to the manufacturer’s instructions. qPCR was performed in a reaction mixture containing cDNA, SYBR Green PCR Master Mix, ddH_2_O, and specific primers on a qTOWER3G (AJ, Viersen, Germany) Real-Time qPCR Detection System. Data were analyzed using the 2^−ΔΔCt^ method. The following primer sequences were used:

*lin-24*: F: 5′-GCAAGGGCTCATGCCAATTC-3′, R: 5′-AAATTGGGTTGGTGGTGAGG-3′;

*lipl-3*: F: 5′-GTGCACTACTGCTCGTGATTC-3′, R: 5′-CATAGTCCAGTCTGTGGATGC-3′.

### 4.5. RNA-Sequencing

For RNA-seq analysis, 1 μg of total RNA was used following the TrueLib mRNA Library Prep Kit for Illumina (ExCell Bio, Shanghai, China) protocol. mRNA was isolated, fragmented, and reverse-transcribed into cDNA. The cDNA was barcoded with multiplex adapters, and a cDNA library was constructed. Libraries were purified using AmpureXP beads, quantified with Qubit (Invitrogen, Waltham, MA, USA), and sequenced on an Illumina HiSeq platform.

### 4.6. RNA-Seq Analysis

Raw RNA-seq data in FASTQ format were mapped to the *C. elegans* genome (WS245) using HTSeq, and read counts were summarized. Count data were imported into R (version 4.2.0) for further analysis. Replicability between samples was evaluated using principal component analysis (PCA) via the prcomp function in R. Differential gene expression analysis was performed using the R/Bioconductor package limma (version 3.48.3) through linear modeling. Transcripts with Fold-Change ≥ 1.5 and *p* < 0.05 were considered significantly differentially expressed genes (DEGs).

### 4.7. Oil Red O Staining

Nematodes were collected in 1.5 mL Eppendorf tubes, centrifuged, and washed three times with M9 buffer. Nematodes were fixed in 4% paraformaldehyde (PFA) for 10 min and stored at −80 °C after quick-freezing in liquid nitrogen. Samples were washed with M9 buffer to remove PFA, stained with Oil Red O (Sigma, Burlington, MA, USA) at room temperature for 30 min, and washed 2–3 times with M9 buffer. Stained nematodes were transferred to agar pads containing 2% agarose and imaged using a standard light microscope (Zeiss, Jena, Germany). Oil Red O staining was quantified using ImageJ 1.8.0 software by calculating the average staining intensity across each nematode’s body.

### 4.8. Lipid Profiling

About 1000 *C. elegans* in each described condition were collected and washed three times with M9 in a 15 mL tube and rotated at room temperature for 30 min to remove intestinal bacteria. After bowel clearance, they were washed twice with M9 and centrifuged. The particles were frozen in liquid nitrogen and then kept at −80 °C until processed. Lipid extraction and liquid chromatography/mass spectrometry (LC/MS) analysis were performed by LipidALL Technologies Limited (www.lipidall.com, accessed on 23 January 2025). The lipids were extracted twice with chloroform/methanol (1:2) and added to the internal standard. LC/MS analysis was performed using a UPLC-QTRAP 6500 PLUS (Sciex, Framingham, MA, USA). Each condition was analyzed with 4 replicates. Unilateral Tukey’s honesty significance difference tests were used for statistical analysis.

### 4.9. Live Nematode Respiration

Determination of oxygen consumption in living nematodes with Limulus reagent by Hippocampal Bioscience XF24 extracellular flux analyzer. About 50 nematodes were collected under each condition and transferred to each hole of the assay plate, leaving four wells as nematode-free controls, all with a final volume of 500 µL M9. At the same time, each probe tube was hydrated in 1 mL of Seahorse correction solution overnight at 37 °C, the instrument was calibrated and cooled to 20 °C, and the analysis plate was loaded. Each well was mixed for 2 min, precipitated for 2 min, and oxygen concentration was measured for 2 min. The oxygen consumption was recalculated and the background value was subtracted to be measured with a blank well. This cycle was repeated 10 times and 10 rate measurements were made on average per well. The plate was ejected, the exact number of nematodes per hole were counted, and the rate was normalized to the number of nematodes.

### 4.10. Transmission Electron Microscopy

Transmission electron microscopy (TEM) was performed as described by our previous studies [58]. Briefly, nematodes were fixed overnight with 0.1 M PB (pH 7.4) containing 3% paraformaldehyde and 0.1% glutaraldehyde at 4 °C, washed with 0.1 M PB four times for 15 min each, and then with 0.1 M glycine in 0.1 M PB for 30 min at 4 °C. After ethanol gradient dehydration, samples were embedded in LR white resin (Sigma, Burlington, MA, USA) and polymerized at 55 °C for 24 h. Ultrathin (100 nm) sections were prepared using an EM UC7 ultramicrotome (Leica Microsystems, Wetzlar, Germany) and loaded onto 200-mesh Ni grids (EMCN). Sections were imaged using a JEM1400PLUS transmission electron microscope (JEOL Ltd., Tokyo, Japan).

### 4.11. Western Blotting

Nematodes were collected and homogenized in liquid nitrogen, then lysed on ice for 30 min in RIPA lysis buffer (20 mM Tris-HCl, 150 mM NaCl, 1% Triton X-100, 0.5% SDS, and protease inhibitors). Total protein concentrations were quantified using a bicinchoninic acid (BCA) assay (BioRAD, Hercules, CA, USA) according to the manufacturer’s protocol. Equal amounts of protein samples were separated by SDS-PAGE and transferred to a PVDF membrane. The membrane was blocked with 3% BSA solution for 1 h at room temperature and incubated with primary antibodies overnight at 4 °C and secondary antibodies for 1 h at room temperature. The primary antibodies used were anti-β-actin (Proteintech, 1:2000 dilution) and anti-GFP (Andes, Hengshui, China, 1:1000 dilution). HRP-conjugated goat anti-rabbit and goat anti-alpaca secondary antibodies (Servicebio and Andes, respectively) were used at 1:5000 dilution.

### 4.12. Statistical Analysis

Data were analyzed using GraphPad Prism 8 software. All experiments were independently repeated at least three times. Survival curves were analyzed using the log-rank test. Statistical significance was determined using parametric Student’s *t*-tests and one-way ANOVA followed by Tukey’s post hoc test. Data are presented as the mean ± standard deviation (SD). *p*-values less than 0.05 were considered statistically significant.

## Data Availability

The data that support the findings of this study are available in the Materials and Methods of this article. All sequencing data generated for this study is available at the National Genomics Data Center with the following accession number: PRJCA029499.

## Supplementary Materials

The following supporting information can be downloaded at: https://www.mdpi.com/article/10.3390/toxins17020072/s1, Table S1: N2-24 h-vs-N2-0 h; Table S2: LIN-24-OE-0 h-vs-N2-OE-0 h; Table S3: N2 24 h-vs-N2 0 h VS LIN-24-OE 0 h vs N2 0 h; Table S4: LIN-24-OE-24 h-vs-N2-24 h; Table S5: LIN-24-OE-24 h-vs-LIN-24-OE-0 h; Table S6: lipidomics of LIN-24-OE 24 h vs N2 24 h; Table S7: LIN-24-OE 24 h vs N2 24 h VS N2 24 h vs N2 0 h; Figure S1: Comparative analysis of differentially expressed genes (DEGs) between LIN-24-OE worms and wild-type (N2) worms under starvation conditions; Figure S2: Analysis of DEGs in LIN-24-OE worms under starvation conditions compared to normal feeding.
