# Supplementary material for: Pore-Forming Protein LIN-24 Enhances Starvation Resilience in Caenorhabditis elegans by Modulating Lipid Metabolism and Mitochondrial Dynamics
Source: Toxins (Basel). 2025 Feb 6;17(2):72. doi: 10.3390/toxins17020072 (PMC11860826; doi:10.3390/toxins17020072)
Supplement: Supplementary file 1 [file toxins-17-00072-s001.zip › Supplementary figures.pdf]

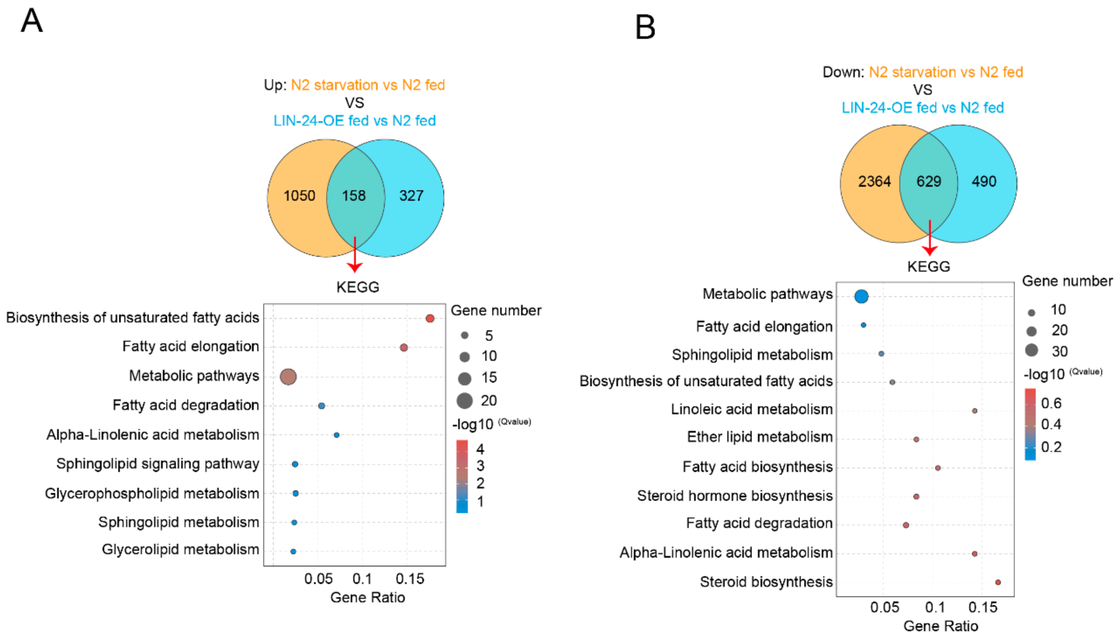

Figure S1. Comparative analysis of differentially expressed genes (DEGs) between LIN-24-OE worms and wild-type (N2) worms under starvation conditions. (A) Venn diagram showing the overlap of upregulated genes between N2 worms under starvation versus feeding conditions (N2 starvation vs. N2 fed) and LIN-24-OE worms under feeding conditions versus wild-type worms (LIN-24-OE fed vs. N2 fed). A total of 158 genes were commonly upregulated. KEGG pathway enrichment analysis of these overlapping genes is displayed below, highlighting their involvement in fatty acid metabolism, unsaturated fatty acid biosynthesis, and other lipid-related pathways. (B) Venn diagram showing the overlap of downregulated genes between the same comparisons as in panel A. A total of 629 genes were commonly downregulated. KEGG pathway enrichment analysis of these overlapping genes reveals significant enrichment in metabolic pathways, fatty acid biosynthesis, and degradation, as well as sphingolipid metabolism.

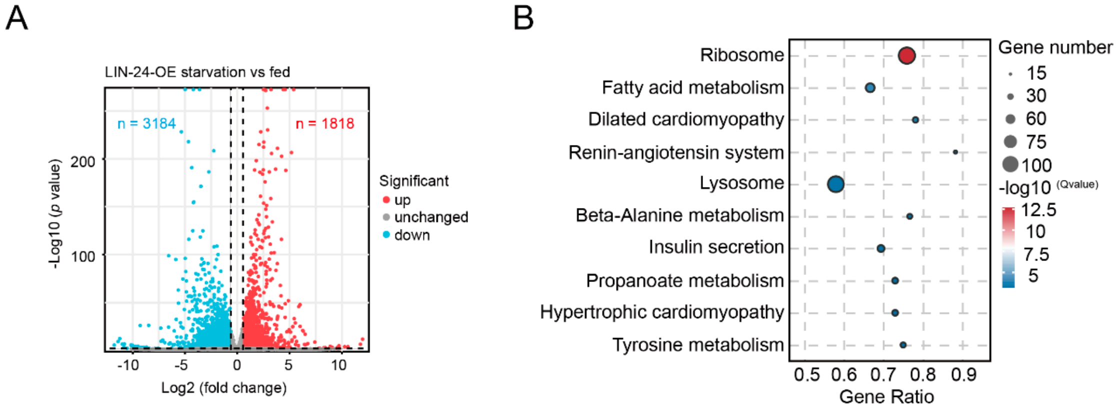

Figure S2. Analysis of DEGs in LIN-24-OE worms under starvation conditions compared to normal feeding. (A) Volcano plot showing significant upregulated (red) and downregulated (blue) genes in LIN-24-OE worms under starvation conditions. The plot illustrates the log2 fold change versus -log10 p-value, with the majority of genes showing significant differential expression. (B) KEGG pathway enrichment analysis of the DEGs, highlighting key pathways involved in lipid metabolism, ribosomal function, and other metabolic processes, with the size of the circles representing the number of genes and the color intensity representing the significance level.
